# Transforming growth factor β receptor 1 is a new candidate prognostic biomarker after acute myocardial infarction

**DOI:** 10.1186/1755-8794-4-83

**Published:** 2011-12-05

**Authors:** Yvan Devaux, Melanie Bousquenaud, Sophie Rodius, Pierre-Yves Marie, Fatiha Maskali, Lu Zhang, Francisco Azuaje, Daniel R Wagner

**Affiliations:** 1 Laboratory of Cardiovascular Research Centre de Recherche Public-Santé, Luxembourg, L-1150, Luxembourg; 2 Nancyclotep Experimental Imaging Platform, Nancy, F-54500, France; 3 Division of Cardiology, Centre Hospitalier, Luxembourg, L-1210, Luxembourg

## Abstract

**Background:**

Prediction of left ventricular (LV) remodeling after acute myocardial infarction (MI) is clinically important and would benefit from the discovery of new biomarkers.

**Methods:**

Blood samples were obtained upon admission in patients with acute ST-elevation MI who underwent primary percutaneous coronary intervention. Messenger RNA was extracted from whole blood cells. LV function was evaluated by echocardiography at 4-months.

**Results:**

In a test cohort of 32 MI patients, integrated analysis of microarrays with a network of protein-protein interactions identified subgroups of genes which predicted LV dysfunction (ejection fraction ≤ 40%) with areas under the receiver operating characteristic curve (AUC) above 0.80. Candidate genes included transforming growth factor beta receptor 1 (TGFBR1). In a validation cohort of 115 MI patients, TGBFR1 was up-regulated in patients with LV dysfunction (P < 0.001) and was associated with LV function at 4-months (P = 0.003). TGFBR1 predicted LV function with an AUC of 0.72, while peak levels of troponin T (TnT) provided an AUC of 0.64. Adding TGFBR1 to the prediction of TnT resulted in a net reclassification index of 8.2%. When added to a mixed clinical model including age, gender and time to reperfusion, TGFBR1 reclassified 17.7% of misclassified patients. TGFB1, the ligand of TGFBR1, was also up-regulated in patients with LV dysfunction (P = 0.004), was associated with LV function (P = 0.006), and provided an AUC of 0.66. In the rat MI model induced by permanent coronary ligation, the TGFB1-TGFBR1 axis was activated in the heart and correlated with the extent of remodeling at 2 months.

**Conclusions:**

We identified TGFBR1 as a new candidate prognostic biomarker after acute MI.

## Background

Left ventricular (LV) remodeling after acute myocardial infarction (MI) sets the stage for the development of heart failure (HF). In spite of modern reperfusion therapies, morbidity and mortality of HF post MI remain elevated, with a 5-year prevalence of 63 to 76% [1,2]. A rapid and accurate prediction of the development of HF after MI would be a major breakthrough since HF is potentially preventable [3].

Several factors determine the magnitude of LV remodeling and dysfunction, including infarct size and other clinical variables such as age, gender and time to reperfusion. These factors have conventionally been used to predict remodeling after acute MI [4]. Circulating biomarkers such as troponins and natriuretic peptides have the potential to improve this prediction and to select patients for new biological or mechanical therapies. However, existing biomarkers are not accurate prognostic indicators of the development of LV remodeling and HF after acute MI.

In recent studies, we have implemented integrated strategies based on the concepts of systems biology to identify new prognostic biomarkers of LV remodeling [5-8]. Approaching LV remodeling with systems-based technologies is a prerequisite to address the complexity of LV remodeling. Some of these studies relied on the assumption that angiogenesis may beneficially affect LV remodeling and participate in cardiac repair. Indeed, intracoronary myocardial contrast echocardiography and magnetic resonance imaging have shown that microvascular perfusion greatly affects LV remodeling [9-11]. However, angiogenesis is certainly not the only regulator of LV remodeling. A transcriptomic profile of angiogenic factors has been revealed [12] and we have reported the capacity of transcriptional networks in blood cells to characterize LV remodeling [8,13].

In the present study, we implemented a combined analysis of transcriptomic profiles of blood cells from MI patients and protein interaction networks of angiogenic proteins to identify new biomarkers of LV remodeling.

## Results

### Patient selection and characteristics of the test cohort

Patients presenting with acute ST-elevation MI, treated with primary percutaneous revascularization, were enrolled in this study. Blood samples were obtained at the time of mechanical reperfusion. A test cohort of two groups of 16 patients selected based on their EF 4 months after MI (Table 1) was used for transcriptomic analyses. One group of patients had a preserved LV systolic function with high EF after MI (> 40%, median 63%, range 45-73), and the other group impaired LV function with low EF (≤ 40%, median 35%, range 20-40). Demographic features of these 2 groups were similar, except for infarct size as indicated by higher levels of TnT and CPK in the low EF group.

**Table 1:** Clinical characteristics.

	Test cohort					Validation cohort	
	**High EF**		**Low EF**		**P value**		
	**(n = 16)**		**(n = 16)**		**(high vs low EF)**	**(n = 115)**	

4-months EF, % (median-range)	63	45-73	35	20-40	0.0003	40	15-86
Age, y (median-range)	56	43-84	68	38-83	0.57	56	32-90
Sex (male, n, %)	13	81%	13	81%	1	98	85%
Body Mass Index (median-range)	28	23-35	26	20-38	0.25	27	19-43

Serum markers (median-range)							
Troponin T (ng/mL)	0.78	0.06-10.69	11.64	1.46-26.1	0.007	4.76	0.03-26.1
CPK (units/L)	762	603-3798	4860	1015-9383	< 0.001	1978	602-9383

Cardiovascular history, n (%)							
Prior MI	2	13%	3	19%	0.64	7	6%
CABG	0	0%	1	6%	0.33	3	3%
PTCA	1	6%	2	13%	0.56	11	10%
Diabetes	4	25%	6	38%	0.46	22	19%
Hypertension	6	38%	7	44%	0.73	47	41%
Hypercholesterolemia	7	44%	9	56%	0.50	45	39%
Tobacco	4	25%	5	31%	0.71	53	46%

Medications, n (%)							
Beta-blockers	16	100%	14	88%	0.16	98	85%
Calcium antagonists	3	19%	0	0%	0.08	0	0%
Nitrates	6	38%	6	38%	1	31	27%
ACE inhibitors	10	63%	10	63%	1	51	44%
Statins	14	88%	14	88%	1	89	77%
Angiotensin receptor inhibitors	2	13%	0	0%	0.16	1	1%

All MI patients had successful mechanical reperfusion and stenting of the infarct artery within 12 hours of chest pain onset. All patients received Aspirin, Clopidogrel, Heparin and Abciximab in the presence of a large thrombus.

ACE: Angiotensin Converting Enzyme; CABG: Coronary Artery Bypass Grafting; EF: Ejection Fraction; PTCA: Percutaneous Transluminal Coronary Angioplasty; MI: Myocardial Infarction.

### Transcriptomic analysis of blood cells

**Figure 1:**
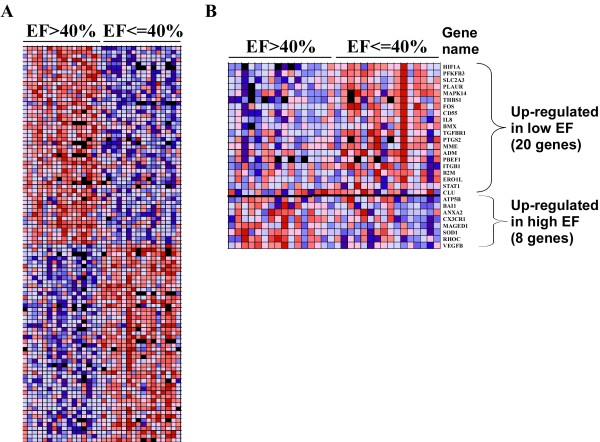
**Gene Set Enrichment Analysis (GSEA) of expression profiles of blood cells from MI patients**. Gene expression profiles of blood cells from two groups of 16 MI patients having either high EF (> 40%) or low EF (≤ 40%) were obtained by 25,000 genes oligonucleotide microarrays. After normalization and filtering steps, expression data were analyzed by GSEA and are visualized by heat-maps. Colors (red, pink, light blue, dark blue) show the range of expression values (high, moderate, low, lowest, respectively). Black boxes denote missing values. (A) Expression ranges of the 50 most differentially expressed genes showing distinct transcriptomic biosignatures between patients with high and low EF. (B) Among the 525 differentially expressed genes between high and low EF patients, 28 genes were involved in angiogenesis according to the Entrez Gene database resource: 20 genes were up regulated in the low EF group and 8 genes were up regulated in the high EF group. A heat-map representing the expression values of these 28 genes is shown.

Gene expression profiles of whole-blood cells were obtained using 25,000 genes microarrays [14]. 525 genes were found differentially expressed between high and low EF patients with a 1.3-fold change threshold. Of note, the 5% threshold for statistical significance was reached for 54% of these genes. 226 genes were up-regulated in the high EF group and 299 were up-regulated in the low EF group. Using gene set enrichment analysis (GSEA), we retrieved the 50 genes most significantly associated with one or the other group of patients. The heat-map drawn with the expression data of these 50 genes shows that LV function assessed 4-months after MI is associated with a specific biosignature of blood cells obtained the day of MI (Figure 1A).

### Angiogenic genes associated with clinical outcome after MI

There is ample evidence that angiogenesis plays a significant role in LV remodeling after MI. We therefore aimed to identify among the 525 genes differentially expressed between high and low EF patients the genes related to angiogenesis. For this purpose, we retrieved from the Entrez Gene database a list of 494 genes known to be related to angiogenesis in humans with the following query: "angiogenesis" AND "homo sapiens". We assigned to these 494 genes the expression values of the test cohort obtained by microarrays. 28 genes were found differentially expressed: 20 genes were up-regulated in the low EF group and 8 genes were up-regulated in the high EF group (Figure 1B).

### Building of a network of protein-protein interactions

**Figure 2:**
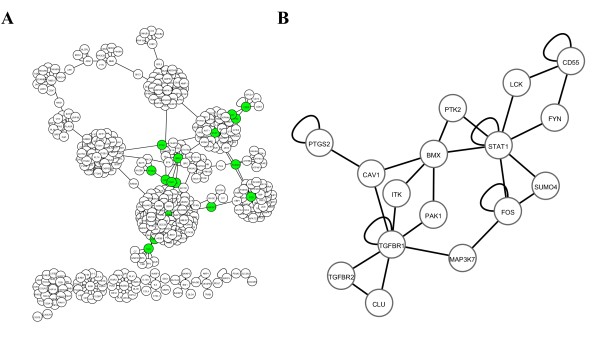
**Protein-protein interaction network**. A global network (A) was built from 441 proteins (nodes) and 458 interactions (edges). Green nodes represents the 16 proteins of cluster 6 used to built the subnetwork (B).

We implemented an interaction network of proteins know to be involved in angiogenesis, a process involved in LV remodeling. For this purpose, we retrieved from the Human Protein Reference Database all known interactions between the 28 proteins encoded by the 28 genes differentially expressed between the 2 groups of patients from the test cohort (Figure 1B). The network built with these interactions is represented in Figure 2A, and contains 441 nodes (proteins) and 458 edges (interactions). It has to be noted that the proteins PBEF1 and PFKFB3 did not have known protein-protein interaction. Then we used MCODE algorithm to identify clusters of interconnected proteins. This was based on the assumption that highly interacting proteins may have important biological functions. Nine clusters from 9 to 35 proteins were identified (Table 2).

**Table 2:** Subsets of angiogenic genes associated with LV function.

Cluster	Protein number	Interaction number	MCODE score*	Genes
1	3	4	1	TGFBR1, TGFBR2, CLU
2	5	7	1	TGFBR1, TGFBR2, CLU, MAP3K7, FOS
3	10	16	1.3	TGFBR1, TGFBR2, CLU, MAP3K7, FOS, SUMO4, STAT1, PTK2, BMX, CAV1
4	12	20	1.42	TGFBR1, TGFBR2, CLU, MAP3K7, FOS, SUMO4, STAT1, PTK2, BMX, CAV1, ITK, PAK1,
5	13	22	1.38	TGFBR1, TGFBR2, CLU, MAP3K7, FOS, SUMO4, STAT1, PTK2, BMX, CAV1, ITK, PAK1, PTGS2
6	16	27	1.37	TGFBR1, TGFBR2, CLU, MAP3K7, FOS, SUMO4, STAT1, PTK2, BMX, CAV1, ITK, PAK1, PTGS2, FYN, LCK, CD55
7	21	34	1.38	TGFBR1, TGFBR2, CLU, MAP3K7, FOS, SUMO4, STAT1, PTK2, BMX, CAV1, ITK, PAK1, PTGS2, FYN, LCK, CD55, ATP5B, SRC, MYOC, ANXA2, PLG
8	28	44	1.32	TGFBR1, TGFBR2, CLU, MAP3K7, FOS, SUMO4, STAT1, PTK2, BMX, CAV1, ITK, PAK1, PTGS2, FYN, LCK, CD55, ATP5B, SRC, MYOC, ANXA2, PLG, SOD1, BCL2, MAPK14, SMAD7, SHC1, MME, ADM
9	35	55	1.34	TGFBR1, TGFBR2, CLU, MAP3K7, FOS, SUMO4, STAT1, PTK2, BMX, CAV1, ITK, PAK1, PTGS2, FYN, LCK, CD55, ATP5B, SRC, MYOC, ANXA2, PLG, SOD1, BCL2, MAPK14, SMAD7, SHC1, MME, ADM, CFH, THBS1, KNG1, PLAUR, PRKCA, RAB25

*MCODE cluster score = density * number of nodes.

### Prognostic performances of clusters of angiogenic genes

To determine the prognostic value of the genes of the clusters identified by network analysis, we used the expression values obtained by microarrays in the two groups of MI patients of the test cohort. To limit our investigations to a reasonable number of genes, we focused on the 16 genes of cluster 6 (Table 2). Of these genes, TGFBR2 and SUMO4 were not detected by microarrays. The sub-network formed by the 16 genes is displayed in Figure 2B. Logistic regression models attributed the best predictive value to a group of 3 genes: TGFBR1, PTK2 and ITK (AUC = 0.89). These 3 genes classified patients into low EF and high EF groups with 87% sensitivity (Table 3).

**Table 3:** Prognostic performances of subsets of angiogenic genes.

Proteins	AUC	True positive rate (%)	False positive rate (%)
TGFBR1, CLU, MAP3K7, FOS, STAT1, PTK2, BMX, CAV1, ITK, PAK1, PTGS2, FYN, LCK, CD55	0.67	62	38
CLU, FOS, ITK, PTGS2, LCK, CD55	0.83	69	31
FOS, LCK, CLU	0.84	69	31
ITK, LCK, CD55	0.82	72	28
ITK, CD55	0.84	75	25
TGFBR1, PTK2, ITK	0.89	87	13

Logistic regression models were used for these analyses. AUC: area under the receiver operating characteristic (ROC) curve. True positive rate (= sensitivity) indicates the percentage of patients correctly classified in the EF group (EF ≤ 40% or EF > 40%). False positive rate (= 1-specificity) indicates the percentage of patients misclassified in the EF group. Optimal classification models are shown; all other combination of genes provided lower classification performances.

### Independent validation

**Figure 3:**
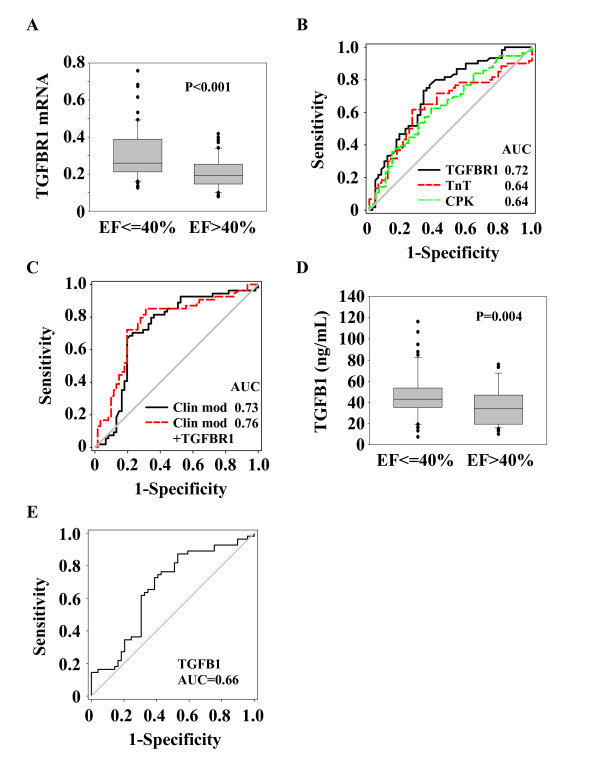
**Expression levels and prognostic values of TGFBR1 and TGFB1**. Blood samples from 115 MI patients obtained upon admission were used to measure TGFR1 mRNA expression in blood cells by quantitative PCR and TGFB1 plasma level by ELISA. TGFBR1 mRNA expression was normalized to SF3A1. (A and D) Box-plots showing that TGFBR1 and TGFB1 are over-expressed in patients with LV dysfunction (EF ≤ 40%) compared to patients with preserved LV function (EF > 40%) assessed at 4-months follow-up. The lower boundary of the box indicates the 25th percentile, the line within the box marks the median, and the upper boundary of the box indicates the 75th percentile. Whiskers (error bars) above and below the box indicate the 90th and 10th percentiles. (B-C-E) ROC curve analysis showing the performance of TGFBR1 and TGFB1 measured at presentation, peak levels of TnT and CPK, and a mixed clinical model (Clin mod; including age, gender and time to reperfusion) to predict LV dysfunction. AUCs are indicated.

To confirm the results obtained from the test cohort, we measured the expression of TGFBR1, PTK2 and ITK by quantitative PCR in blood cells from a validation cohort of 115 acute MI patients. Demographic features of these patients are shown in Table 1. As for the test cohort, blood samples were obtained at presentation and follow-up was performed at 4-months by echocardiography. Of the 3 candidate genes, TGFBR1 was the most robustly associated with LV function. PTK2 and ITK were not significantly correlated with the EF (data not shown). TGFBR1 expression was inversely correlated with the EF (r = -0.28, P = 0.003) and was higher in patients with LV dysfunction (Figure 3A). Linear regression analyses attested that TGFBR1 was associated with LV dysfunction at 4-months (P = 0.003). The serum markers creatine phosphokinase and troponin T predicted TGFBR1 expression (P = 0.03 and P = 0.003 by linear regression, respectively), suggesting that TGFBR1 is responsive to infarct size. All together, these results show that TGFBR1 expression is associated with LV function in an independent cohort.

### Prognostic performance of TGFBR1

The prognostic value of TGFBR1 in the cohort of 115 acute MI patients was first evaluated using ROC curve analysis, which reported an AUC of 0.72 (Figure 3B). As a comparison, peak levels of TnT and CPK provided an AUC of 0.64 (Figure 3B). CPK or troponin T did not improve the prognostic value of TGFBR1 (not shown), suggesting that this value is independent of infarct size. Reclassification analyses reported a NRI of 8.2% when TGFBR1 was added to TnT prediction (Table 4).

**Table 4:** Reclassification performance of TGFBR1 over TnT.

	TnT			TnT + TGFBR1			
**Class**	**EF > 40%**	**EF ≤ 40%**	**Correctly classified (%)**	**EF > 40%**	**EF ≤ 40%**	**Correctly classified (%)**	**NRI (%)**

**EF > 40%**	**32**	23	59.3	**38**	20	70.4	
**EF ≤ 40%**	22	**38**	62.2	16	**41**	67.2	
Total	54	61	60.1	54	61	68.7	8.2

Correct classifications appear in bold. NRI: net reclassification index.

In the validation cohort, 7 patients (6%) had a previous MI (Table 1). In the 108 patients who presented with a first MI, the predictive value of TGFBR1 had an AUC of 0.72, identical to the AUC reported for the whole validation cohort (Figure 3B). Therefore, although it is expected that a population of patients with first infarcts would better reflect post-MI LV remodeling, the presence of few patients with a second infarct did not affect the predictive value of TGFBR1.

We next investigated the additive value of TGFBR1 to a mixed clinical model including age, gender and time to reperfusion, all known to determine the extent of LV remodeling. We observed significant correlations between the EF measured at 4-months and each of these parameters, patients of older age (R = -0.35, P = 0.0002), females (R = 0.20, P = 0.02), and presenting late after chest pain onset (R = -0.26, P = 0.007) having the highest risk of impaired EF (EF ≤ 40%). By multiple logistic regression, however, only age was a significant predictor of impaired EF (P < 0.001). The clinical model predicted LV function with an AUC of 0.73 (Figure 3C), comparable to the AUC provided by TGFBR1 alone (AUC = 0.72, Figure 3B). Adding TGFBR1 to the clinical model increased its AUC to 0.76 (Figure 3C). Reclassification analyses attested that TGFBR1 was able to reclassify 17.7% of patients misclassified by the clinical model (Table 5).

**Table 5:** Reclassification performance of TGFBR1 over a clinical model including age, gender and time to reperfusion.

	Clinical model			Clinical model + TGFBR1			
**Class**	**EF > 40%**	**EF ≤ 40%**	**Correctly classified (%)**	**EF > 40%**	**EF ≤ 40%**	**Correctly classified (%)**	**NRI (%)**

**EF > 40%**	**43**	22	79.6	**43**	17	79.6	
**EF ≤ 40%**	11	**39**	63.9	11	**44**	72.1	
Total	54	61	71.3	54	61	75.6	17.7

Correct classifications appear in bold. NRI: net reclassification index.

Together, these data confer a significant prognostic value to TGFBR1.

### Prognostic performance of TGFB1

We then evaluated the ability of TGFB1, the ligand of TGFBR1, to predict LV dysfunction. TGFB1 was measured in the plasma obtained at presentation in the 115 patients of the validation cohort. As observed for TGFBR1, TGFB1 was inversely correlated with EF (r = -0.20, P = 0.04) and was over-expressed in patients with LV dysfunction (Figure 3D). Linear regression revealed a significant association between TGFB1 and LV dysfunction (P = 0.006) and ROC curves reported a modest prognostic value with an AUC of 0.66 (Figure 3E). Adding TGFB1 to TGFBR1 did not result in a statistically detectable improvement in prediction (not shown). Therefore, TGFB1 is also associated with LV function but does not provide an additive value to TGFBR1.

### The TGFB1-TGFBR1 axis in the heart

**Figure 4:**
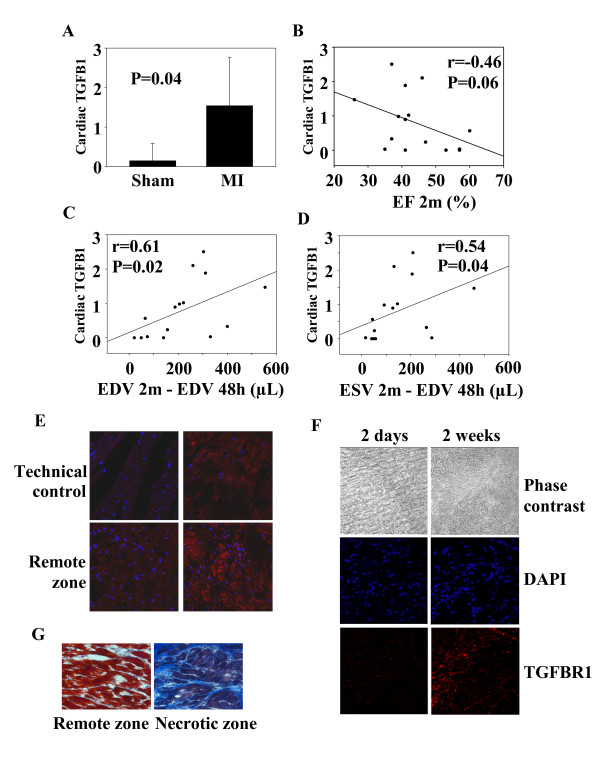
**The TGFB1-TGFBR1 axis in rats after MI**. 17 rats were subjected to MI through permanent ligation of the LAD coronary artery. 4 rats were sham-operated. Rats were assessed by PET to determine LV volumes and EF 48 hours and 2 months after ligation. Rats were sacrificed after 2 days (n = 3), 2 weeks (n = 3) or 2 months (n = 11). At sacrifice, hearts were harvested. (A) Total TGFB1 measured by ELISA in cardiac samples is up-regulated 2 months after MI. Data are expressed in pg of TGFB1 per μg of total cardiac proteins. Shown are mean ± 95% CI (n = 4 for sham, n = 11 for MI). (B) TGFB1 expression in the heart mildly correlates with 2-months EF. (C-D) TGFB1 expression correlates with the variations of LV end-diastolic and end-systolic volumes between 48 hours and 2 months. Correlation coefficients and P values are indicated. (E) Immunohistochemical analysis of TGFBR1 expression in cardiac slices. The technical control without anti TGFBR1 antibody attests for the specificity of the detection. TGFBR1 staining appears in red colour and nuclei appear in blue. TGFBR1 expression is up-regulated in the border zone of MI rats. Representative pictures of sham-operated rats (upper panels) and MI rats (lower panels) are shown. (F) TGFBR1 expression in the border zone 2 days and 2 weeks after MI. TGFBR1 expression is up-regulated in the border zone 2 weeks after MI. Representative pictures are shown. (G) Masson trichrome staining showing collagen deposition and fibrosis in the necrotic zone of the heart 2 months after MI. Representative pictures from a MI rat are shown.

To investigate the physiological relevance of our findings, we subjected rats to LAD-occlusion, a standard animal model of MI. The amount of total TGFB1 in the heart was increased 2 months after MI (Figure 4A). However, the amount of activated TGFB1 was not increased (not shown), suggesting that the up-regulation of TGFB1 expression in the heart of MI animals reflects an increase of the latent form. Total TGFB1 expression did not significantly correlate with LV function, as assessed by the EF at 2-months (Figure 4B). Interestingly, there was a positive correlation between TGBF1 and LV remodeling, as assessed by the variation of end-diastolic and end-systolic volumes between 48 hours and 2 months after MI. Rats with the largest degree of remodeling (increased LV volumes) had the highest levels of TGFB1 (Figure 4C-D).

Faintly expressed in the heart of healthy animals (sham), TGFBR1 was up-regulated in the border zone but not in the remote zone 2-months after MI (Figure 4E). TGFBR1 was highly expressed 2 weeks after MI, but not after 2 days (Figure 4F). Trichrome Masson staining showed the presence of collagen deposition and fibrosis in the necrotic part of the heart after 2 months (Figure 4G). Therefore, the TGFB1-TGFBR1 axis is activated in cardiac tissue after experimental MI in rats and correlates with LV remodeling.

## Discussion

Based on the concepts of systems biology, this study was designed to identify new prognostic biomarkers after acute MI. First, using a test cohort of 32 patients, we observed that LV dysfunction after MI (4-months EF < 40%) had a biosignature in blood cells in the acute phase. Among the genes associated with LV dysfunction, 28 were linked to angiogenesis, which is known to play a key role in cardiac repair. Then, using clustering analysis of a network of protein-protein interactions built with these 28 angiogenic genes, we isolated a group of 3 genes -TGFBR1, PTK2, ITK- which predicted LV function with and AUC of 0.89. In an independent validation cohort of 115 MI patients, TGFBR1 was found to have a prognostic value which may become clinically useful. Finally, we showed that the TGFB1-TGFBR1 is activated after MI in rats and correlates with the extent of remodeling.

Left ventricular remodeling is a highly complex phenomenon involving diverse biological processes, such as inflammation, regulation of extracellular matrix turnover, fibrosis, cell death, and angiogenesis. Such complex diseases can be approached with systems-based concepts. We chose angiogenesis to filter the relatively high number of genes (525) found to be differentially expressed between patients with or without LV dysfunction. It is clear that other family of genes involved in remodeling may also be worth studying. It is expected that genes involved in multiple biological pathways or sharing many interactions with other genes may be more susceptible to play significant roles in remodeling and have a prognostic value. Accordingly, TGFBR1 was a node in the network of 16 angiogenic proteins shown in Figure 2B and was connected to 6 other proteins, suggesting a central role in angiogenesis. A more global approach, which could be used for future studies, would be to widen the search for biomarkers to all pathways known to play a role in LV remodeling, and not to restrain to a specific pathway such as angiogenesis.

Transcriptional profiling has emerged as an interesting tool to study cardiovascular diseases and ultimately to personalize therapeutic strategies [15]. A key feature of our study is the use of readily available blood cells, rather than cardiac biopsies. While it may be argued that transcriptomic analysis of cardiac tissues would more accurately reflect the myocardial response to MI, it is accepted that several cardiovascular conditions including coronary artery disease [16] and chronic HF [17] are characterized by specific transcriptomic biosignatures in blood cells. Our study shows for the first time that TGFBR1 expression level in blood cells obtained at presentation in acute MI patients has prognostic value for long-term LV remodeling.

In addition, we have observed an association between TGFB1, which has prognostic values for remodeling and hypertrophy in patients with hypertension and aortic stenosis [18,19], and LV function. This association was not as robust as TGFBR1, and the prognostic value of TGFBR1 was not found to be improved by TGFB1 determination. Interestingly, TGFBR1 added some value to the prediction of TnT, a marker of infarct size known to affect LV remodeling. The reclassification of 8.2% of patients misclassified by TnT is clinically relevant. Similarly, the reclassification of 17.7% of patients misclassified by a standard risk factor model based on age, gender and time to reperfusion, is pertinent.

The integration of multiple biomarkers is thought to improve the estimation of the course of cardiovascular diseases [20], although this assumption has been questioned [21]. Heidecker and coworkers reported that a group of 45 genes identified from endomyocardial biopsies predicted 5-year outcome of new onset HF patients with a sensitivity of 74% and a specificity of 90% [22]. More recently, a panel of 5 plasma proteins involved in extracellular matrix turnover was demonstrated to be a more accurate predictor of LV hypertrophy than any single biomarker [23]. Alternatively, combining biomarkers representing diverse disease mechanisms has also been shown to add incremental risk stratification value in patients with non ST-elevation acute coronary syndrome [24]. Our data showing that TGFBR1 has an additive value to traditional markers are consistent with the concept that multiplication of biomarkers may be clinically useful.

More than 80% of the patients enrolled in this study were males. The low number of females enrolled (6 in the test cohort and 17 in the validation cohort) prevented us from investigating whether gender contributes to the prognostic value of TGFBR1. However, TGFBR1 expression was not different between women and men (not shown), suggesting that gender may not influence the prognostic value of TGFBR1.

Activation of the TGFB1-TGFBR1 pathway is mainly associated with stimulation of fibrosis, although it also affects LV hypertrophy, matrix metabolism, inflammation, and angiogenesis [25-27]. After MI, this pathway triggers the switch from inflammation to fibrosis [28-30]. TGFBR1 expression has been reported to be higher in the infarcted region of pig hearts after permanent coronary ligation compared to the remote region [31]. Our data in rats confirm and extend these observations. It is believed that biomarkers should reflect the pathophysiology, and our experiments in rats provide valuable proof for this. Inhibition of TGFBR1 activity by orally active specific inhibitors [28,32] or competitive inhibition of TGFB1 by a soluble form of TGFBR2 [33] dampens cardiac remodeling after MI. Other strategies to block TGFB, which have been recently reviewed [34], may also be tested to limit cardiac remodeling

Endothelial progenitor cells participate in cardiac revascularization and healing after acute MI. However, considering their extremely low frequency in the circulation, these cells are certainly not the main source of TGBFR1 signal measured by microarrays. Circulating monocytes-macrophages may most probably account for the majority of TGFBR1 expression. Interestingly, these cells secrete many cytokines, growth factors and fibrotic factors which regulate LV remodeling and cardiac healing. The higher expression of TGFBR1 in patients with low EF is consistent with the more robust activation of inflammation observed in these patients, as attested by higher white blood cells counts (not shown). Whole blood cells profiling can be affected by shifts in leukocyte populations. However, the proportions of circulating monocytes were comparable between patients with low EF and patients with high EF, suggesting that this confounding factor did not importantly affect our results. It would be interesting to accurately determine which cell type(s) express TGFBR1 in the heart, and whether TGFBR1 expression is regulated in cells involved in cardiac healing. The animal model of coronary artery ligation used in the animal study is a valuable tool to answer these questions.

The main limitation of the present study relies in the small number of patients enrolled. This is particularly relevant for the test cohort used in microarray experiments. Nevertheless, using a different and quantitative technique, we were able to validate our findings from this small cohort in an independent cohort of more than 100 patients. The relevance of our findings depends on the confirmation of the prognostic value of TGFBR1 in larger patient populations. In addition, all human blood samples were collected at presentation and measurement of TGFBR1 expression at different time-points after acute MI could provide valuable informations. Finally, mutations in TGFBR1 gene, known to affect vascular integrity in Marfan and Loeys-Dietz syndromes [35,36], have to be taken into consideration for the design of probes to measure TGFBR1 expression and for the design of specific therapeutic inhibitors.

## Conclusions

We have shown that TGFBR1 expression in blood cells of acute MI patients is associated with LV remodeling. If confirmed in independent studies, this new biomarker may become clinically useful to identify patients with acute MI who are at risk of adverse outcome and may benefit from novel therapies.

## Methods

### Patients

Patients with acute MI were enrolled in a national MI registry and treated with primary percutaneous coronary intervention. Acute MI was defined by the presence of chest pain < 12 hours, significant ST elevation (> 1 mm), completely occluded major coronary artery (TIMI O flow in LAD, CX or RCA), peak (≈ 24 hours) creatine phosphokinase (CPK) level > 600 units/L and peak troponin T (TnT) level > 0.03 ng/mL. Blood samples obtained shortly (5 min) after mechanical reperfusion, via an arterial catheter and into PAXgene™ tubes (BD Biosciences, Erembodegem, Belgium), were used for determination of TGFBR1 expression. TGFB1 was measured in plasma extracted from citrated tubes collected after mechanical reperfusion. Time to reperfusion was recorded by dedicated nurses as the delay between chest pain onset evaluated by the patient and presentation. CPK activity was assessed with a Roche IFCC method on a Cobas c501 instrument (Roche, Prophac, Luxembourg). TnT was assessed with a 4^th ^generation assay from Roche performed on a Cobas e601 equipment (Roche). TGF-B1 was measured with the human TGF-B1 ELISA kit (ref DB100B, R&D Systems, Oxon, UK). LV function was assessed by echocardiography 4 months after MI, and LV remodeling was defined by a LV ejection fraction (EF) ≤ 40%. The protocol has been approved by the local ethics committee (National committee of ethics in research of the Grand-Duchy of Luxembourg) and informed consent has been obtained from all subjects.

### Microarrays

Transcriptomic analysis of blood cells was performed as previously described [7]. Total RNA was extracted from whole blood cells of MI patients using the PAXgene™ blood RNA kit (Qiagen, Venlo, Netherlands). A second purification and concentration step was performed with the RNeasy^® ^MinElute™ kit (Qiagen). RNA quantity was measured using the ND-1000 spectrophotometer (NanoDrop^® ^Technologies, Wilmington, USA). RNA quality was assessed using the 2100 Bioanalyzer^® ^apparatus (Agilent Technologies, Massy, France) with the RNA 6000 Nano chips. Only high quality RNA (OD_260_/OD_280 _> 1.9 and OD_260_/OD_230 _> 1.7) and un-degraded RNA was considered for further analysis. A common reference RNA (Universal Human Reference RNA, Stratagene Europe, Amsterdam, The Netherlands) was used in conjunction with patient's RNA to provide an internal reference standard for comparisons of relative gene expression levels across arrays. Messenger RNAs were amplified using the Amino Allyl MessageAmp™ kit (Ambion^®^, Cambridgeshire, United Kingdom), starting with one μg of total RNA. Five μg of each amino allyl aRNA were labeled with Cy3 or Cy5 (Amersham, Buckinghamshire, United Kingdom). Dye coupling to RNA was measured using the ND-1000 NanoDrop^® ^spectrophotometer. Coupling yield > 5% was a prerequisite for further analysis. 750 ng of each amino allyl aRNA labeled Cy3 or Cy5 (reference RNA or patient RNA) were combined and hybridized on oligonucleotide microarrays representing 25,000 genes [14]. Four replicates and a dye-swap were performed. Scanning was performed with an Axon 4000B scanner and data were acquired with the GenePix Pro 6^® ^software (Molecular Devices, Berks, UK). Spot finding and raw data quantification were performed using the MAIA^® ^freeware. A Lowess non linear normalization was performed and genes that were not present in at least 3 microarrays of 4 were filtered out. Data are available at the Gene Expression Omnibus database (http://www.ncbi.nlm.nih.gov/geo/) under the accession number GSE11947.

Before statistical analysis, genes not present in at least 50% of the patients were filtered out. Supervised analysis was performed using the Significance Analysis of Microarrays software using 2-class unpaired test and 100 permutations. Heat maps were drawn using the Gene Set Enrichment Analysis (GSEA) software [37].

### Quantitative PCR

The expression of mRNAs in blood cells of MI patients obtained upon admission was determined by quantitative PCR. Total RNA was extracted from blood cells collected in PAXgene^® ^blood RNA tubes. PCR was performed using a CFX96 device and the IQ™ SYBR^® ^Green Supermix (BioRad, Nazareth, Belgium). SF3A1 was used for normalization.

### Network of protein-protein interactions

Genes known to be associated with angiogenesis in humans were retrieved from the Entrez-Gene database [38] with the query: "angiogenesis" AND "homo sapiens". The Human Protein Reference Database (release 9) [39] was then interrogated to identify all annotated protein-protein interactions associated with these genes. These interactions were used as inputs to build a network, which was visualized and analyzed with Cytoscape [40]. Clusters of highly interconnected proteins were identified by the MCODE plug-in network clustering algorithm [41].

### In vivo experiments and biochemical analyses

Myocardial infarction was induced in 17 anesthetized Wistar rats (Charles Rivers Laboratories; 8-11 weeks; 310-380 g) by permanent occlusion of the left anterior descending (LAD) coronary artery as previously described [42,43]. An additional 4 rats were sham-operated. All rats were monitored *in vivo *48 hours after surgery and after 2 months with the use of a high resolution dedicated small animal positron emission tomography (PET) system with^18^F-FDG (IBA, Nancy, France) as a tracer and a premedication by acipimox, as previously described [44]. LV end-diastolic volume (LVEDV) was obtained on contiguous gated short-axis slices by the Quantitative Gated SPECT software [45]. The extent of remodeling was assessed by the change in LVEDV between 48-hours and 2-months after surgery (Δ LVEDV). At sacrifice, blood samples were collected, hearts were harvested, snap frozen and embedded in OCT. Parts of cardiac tissue were rinsed in NaCl and stored at -80°C for protein extraction. These experiments were conducted in accordance with the regulations of the Animal Welfare Act of the National Institutes of Health Guide for the Care and Use of Laboratory Animals (NIH Publication No. 85-23, revised 1996) and protocols were approved by local Ethics Committee and by the Regional Veterinary Department.

For immunohistochemistry and collagen-specific Masson Trichrome staining (Dako, Leuven, Belgium), 8 μm frozen heart sections were generated. Primary antibody used was a rabbit polyclonal anti TGFBR1 antibody (Abcam, Cambridge, UK). Alexa Fluor^®^568-coupled donkey anti-rabbit antibodies (Jackson ImmunoResearch, UK) were used as secondary antibodies. Images were recorded on a confocal microscope (Zeiss Laser Scanning Microscope LSM 510) with a 40× objective using the LSM 510 META software. DAPI (blue) staining was used to reveal nuclei.

The TGFB1 ELISA kit (ref MB100B, R&D Systems) was used to measure TGFB1 in cardiac tissue. 50 μg of total proteins from each heart were assayed. This assay detects activated TGFB1 with a sensitivity of 4.6 pg/mL. To measure total TGFB1, latent TGFB1 is activated by acidification with hydrochloric acid prior to assay. Coefficient of variation intra-assay is < 4% and coefficient inter-assay is < 9%.

### Statistical analysis

Comparisons between means of two groups were performed with the Mann-Whitney test. Categorical variables were compared using the Fisher exact test. Correlation between biomarker levels and the EF class was estimated with the Spearman test. All tests were two-tailed and were preceded by a normality test (Shapiro-Wilk). A *P *value < 0.05 was considered statistically significant. The SigmaPlot (v. 11.0) was used to carry-out statistical tests.

Prognostic performances of single genes were evaluated using receiver operating characteristic (ROC) curves and the area under the ROC curves (AUC), as well as linear regression models. Logistic regression models with ridge parameter of 1.0E-8 used to evaluate the predictive performances of multiple markers were implemented and tested using 10-fold cross-validation on the Weka (v. 3.4) data mining platform [46].

Reclassification analyses have been performed to evaluate the additive value of TGFBR1 to TnT and to a mixed clinical model. The net reclassification index (NRI) was calculated as the difference in proportions moving up and down among cases and controls: [Pr(up|case)-Pr(down|case)]-[Pr(up|control)-Pr(down|control)].

## Competing interests

The authors declare that they have no competing interests.

## Authors' contributions

YD conceived the study and prepared the manuscript; MB performed in vivo experiments; SR performed in vitro experiments; PYM and FM participated in animal experiments; LZ analyzed microarray data; FA and DRW participated in study design and revised the manuscript. All authors have read and approved the final manuscript.

## Pre-publication history

The pre-publication history for this paper can be accessed here:

http://www.biomedcentral.com/1755-8794/4/83/prepub
